# A collision tumor of nasopharyngeal carcinoma and primary mantle cell lymphoma in the nasopharynx: a case report and review of the literature

**DOI:** 10.1186/s12903-023-03415-y

**Published:** 2023-09-17

**Authors:** Meng Jiang, Xiao-ping Yuan, Hong Zhang, Chuang-quan Li, Yong-lin Mao, Wei-liang Chen

**Affiliations:** 1 School of Medicine, Sun Yat-Sen University, Shenzhen, 518107 People’s Republic of China; 2 Department of Radiology, Sun Yat-Sen Memorial Hospital, Sun Yat-Sen University, Guangzhou, 510120 People’s Republic of China; 3 Department of Nuclear Medicine, Sun Yat-Sen Memorial Hospital, Sun Yat-Sen University, Guangzhou, 510120 People’s Republic of China; 4 Department of Oncology, Sun Yat-Sen Memorial Hospital, Sun Yat-Sen University, Guangzhou, 510120 People’s Republic of China; 5 Department of Oral and Maxillofacal Surgery, Sun Yat-Sen Memorial Hospital, Sun Yat-Sen University, Guangzhou, 510120 People’s Republic of China

**Keywords:** Collision tumor, Nasopharyngeal carcinoma, Primary mantle cell lymphoma, MRI, PET-CT, Case report

## Abstract

**Background:**

Nasopharyngeal carcinoma (NPC) is more common in men aged 40 to 59, and radiotherapy is an effective treatment. Nasopharyngeal lymphoma (NPL) is rare, and the coexistence of nasopharyngeal mantle cell lymphoma (MCL) and NPC is even rarer. A collision tumor is a rare type of tumor that refers to two or more different tumors occurring in the same organ. No reports to date have described a collision tumor of NPC and MCL occurring within the same nasopharyngeal mass. We herein report the successful treatment of a unique case of synchronous coexistence of NPC and MCL occurring in the nasopharynx of a Chinese man.

**Case presentation:**

A 58-year-old man presented with a 5-month history of swallowing discomfort. Biopsy was performed under nasopharyngeal endoscopy, and histopathology revealed NPC. Magnetic resonance imaging revealed lesions in the nasopharynx, oropharynx, and tonsils, as well as enlarged lymph nodes in the parotid gland, posterior ear, and neck. This may be a synchronous dual primary tumor coexisting with NPC and NPL. Pathology consultation confirmed that the biopsy specimen of the nasopharynx was a collision tumor of NPC and MCL. Positron emission tomography computed tomography (PET-CT) revealed thickening of the posterior wall of the nasopharynx, which was considered NPC with lymphoma. The enlargement of the pharyngeal lymph ring and multiple hypermetabolic lymph nodes were evaluated as lymphoma infiltration. The patient received two courses of R-CHOP chemotherapy (rituximab, cyclophosphamide, doxorubicin, vincristine, and prednisone) followed by head and neck radiotherapy. At the time of this writing, he had remained alive without recurrence for 61 months since the initial treatment and was still undergoing follow-up.

**Conclusions:**

It is very important to correctly recognize collision tumors. Magnetic resonance imaging helps identify different components of collision tumors. Pathological examination helps to confirm the diagnosis. Histological examination reveals different components, and PET-CT can help determine the extent of the lesion. Dose-adjusted chemotherapy combined with radiotherapy may have promising herapeutic effects, but additional case studies are needed to confirm.

## Introduction

According to data from the International Agency for Research on Cancer Research, there were approximately 133,354 newly diagnosed cases of nasopharyngeal carcinoma (NPC) in 2020, accounting for only 0.7% of all cancers diagnosed that year [1]. Nevertheless, the global distribution of NPC is highly imbalanced, with over 70% of new cases occurring in East and Southeast Asia. The global age standardization rate is 2.2/100,000 for males and 0.8/100,000 for females [1]. Mantle cell lymphoma (MCL), which makes up approximately 5–10% of all lymphomas, is a subtype of B-cell lymphoma derived from CD5-positive, antigen-naïve, pre-germinal center B cells within the mantle zone that surrounds normal germinal center follicles [2]. Primary MCL involving the nasopharynx is extremely rare [2, 3]. Two or more distinct tumors of different cell lineages that independently occur in the same space or organ and combine to form one mass are defined as collision tumors [4]. Collision tumors of carcinoma and lymphoma often occur in the skin [4] and gastrointestinal tract [5]. To the best of our knowledge, to date, there have been no reports in the literature that NPC and primary MCL occur in the same location within the nasopharynx. We herein report the successful treatment of a collision tumor of NPC and primary MCL in the nasopharynx.

## Case presentation

A 58-year-old Chinese man had a 5-month history of swallowing discomfort. Nasopharyngeal endoscopic biopsy of nasopharyngeal lesions confirmed by histopathological examination as nasopharyngeal carcinoma (NPC). The patient was subsequently referred to the Department of Stomatology of Sun Yat-sen Memorial Hospital on 19 April 2018 for further diagnosis and treatment. He reported no spontaneous pain or tenderness in the oropharynx or nasal cavity. He also had no numbness, bleeding, fever, or weight loss. The patient smoked an average of two cigarettes per day for 40 years. He had no history of alcohol abuse or family history of NPC.

Physical examination revealed facial symmetry, a soft neck, and smooth movement. Two enlarged lymph nodes with a diameter of 1.0 and 1.5 cm, respectively, were palpated at the posterior lower pole of the bilateral parotid glands, with a medium to hard texture and clear boundaries. Two other enlarged lymph nodes with a diameter of 3.0 and 2.0 cm, respectively, were palpable on both sides of the jaw; these lymph nodes exhibited moderate mobility without tenderness or adhesion to surrounding tissues. Moreover, an enlarged lymph node with a diameter of 1.0 cm, was palpated under the chin. Blood examination revealed a white blood cell (WBC) count of 6.48 × 10^9^/L (reference range, 3.50–9.50 × 10^9^/L). Red cell count, hemoglobin concentration, platelet count, lymphocyte proportion and count, the serum concentration of alanine aminotransferase, aspartate aminotransferase, total protein, albumin, unsaturated iron binding capacity, carbohydrate antigen 72–4, carbohydrate antigen 125, carbohydrate antigen 19–9, alpha-fetoprotein, and carcinoembryonic antigen were showed in Table 1. Lactate dehydrogenase (LDH) concentration was 141 U/L (reference range, 108–252 U/L). Blood tests for syphilis, hepatitis B virus, hepatitis C virus and human immunodeficiency virus (HIV) were also negative.

**Table 1:** Laboratory Data

Variable	Reference Range, Adults	On Initial Evaluation	After the First Chemotherapy	After the Second Chemotherapy
Red cell count (10^12^/L)	4.30–5.80	5.75	5.46	5.82
Hemoglobin ( g/L)	130–175	125	118	124
Platelet count (10^9^/L)	125–350	253	257	292
Lymphocyte proportion (%)	20–50	32.3	31.2	32.6
Lymphocyte count (10^9^/L)	1.1–3.2	2.09	1.74	1.7
Alanine aminotransferase (U/L)	9–50	17	15	11
Aspartate aminotransferase (U/L)	15–40	16	14	15
Glucose(mmol/L)	3.9–5.6	4.7	4.3	4.6
Total cholesterol(mmol/L)	2.90-6.00	4.15	2.66	4.60
Triglyceride (mmol/L)	0.31–2.30	0.75	1.29	1.26
High density lipoprotein(mmol/L)	0.80–1.96	0.97	0.70	1.01
Low density lipoprotein(mmol/L)	1.30–3.60	2.64	1.53	2.86
Prealbumin (g/L)	0.18–0.40	0.30	0.25	0.30
Total protein (g/L)	65.0–85.0	67.4	67.5	67.1
Albumin(g/L)	40.0–55.0	39.3	36.9	37.2
Globulin(g/L)	20.0–40.0	28.1	30.6	29.9
Albumin/globulin ratio	1.2–2.4	1.4	1.2	1.2
Serum iron(μmol/L)	7.0–32.0	26.7	9.1	11.9
Total iron binding force(μmol/L)	45.0–75.0	50.2	59.0	50.8
Unsaturated iron bonding force(μmol/L)	31.0–51.0	23.5	49.9	38.9
Transferrin(g/L)	1.90–3.80	2.34	2.25	2.16
Carcinoembryonic antigen (ng/ml)	≤ 5	3.3		
Alpha-fetoprotein (ng/ml)	≤ 25	2.06		
Carbohydrate antigen 72 − 4 (U/ml)	≤ 7	1.4		
Carbohydrate antigen 125 (U/ml)	≤ 35	6.3		
Carbohydrate antigen 19 − 9 (U/ml)	≤ 34	4.3		

**Fig. 1:**
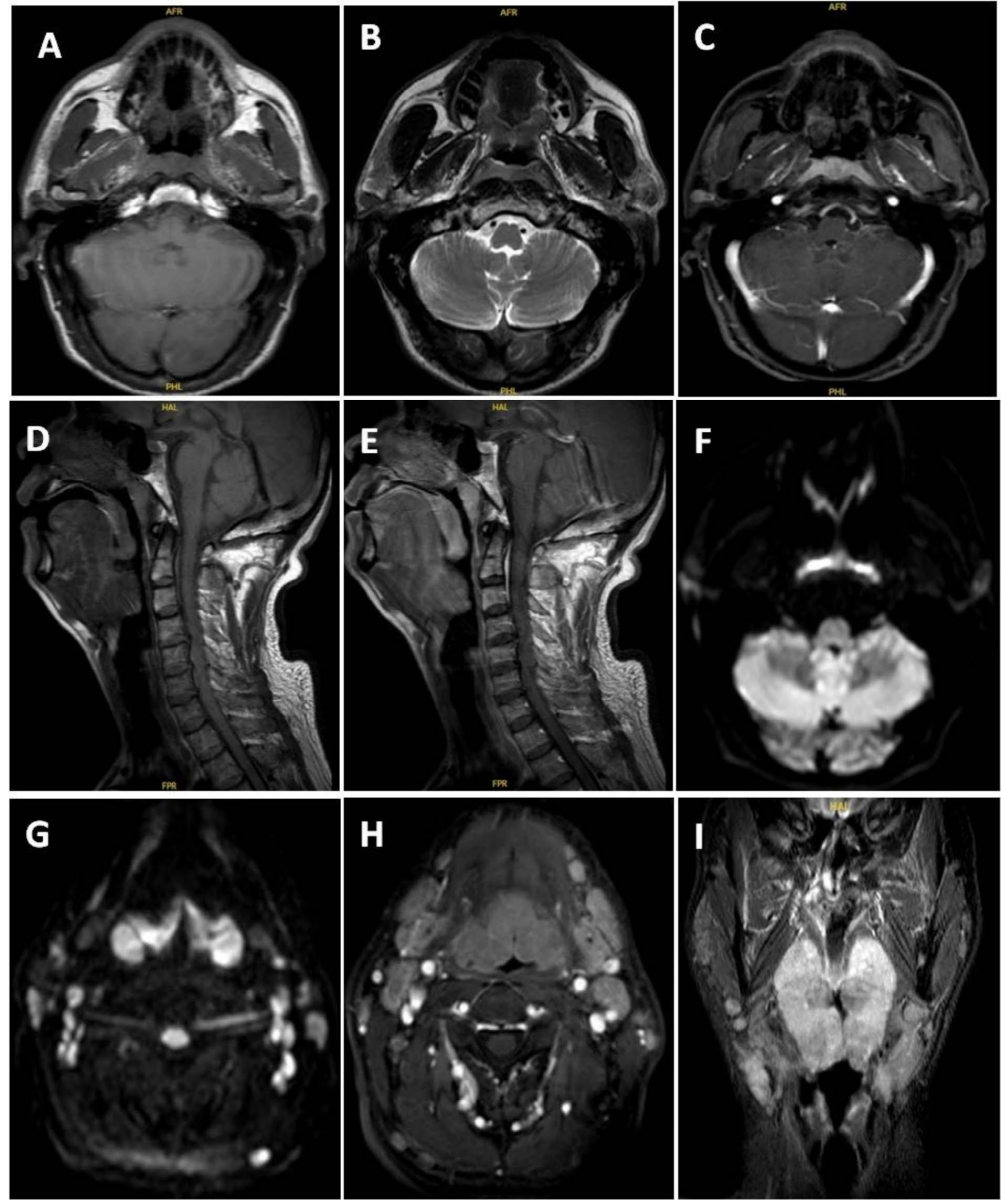
Nasopharyngeal plain and enhanced magnetic resonance imaging. **A**. Cross-sectional T1-weighted imaging (T1WI) showed thickening of the nasopharynx. **B**. Cross-sectional T2-weighted imaging (T2WI) showed thickening of the nasopharynx (the signal was uneven and exhibited boundedness, with a range of approximately 7 × 5 mm). **C**. Cross-sectional T1WI with contrast showed uneven and significant enhancement in the thickened area of the nasopharynx. **D**. Sagittal T1WI showed thickening of the nasopharynx and pharyngeal tonsils. **E**. Sagittal T1WI with contrast showed thickening with noticeable enhancement and enhancement of the nasopharynx and pharyngeal tonsils. **F**. Diffusion-weighted imaging showed limited diffusion in the nasopharyngeal lesion area. **G**. Diffusion-weighted imaging showed limited diffusion in the pharyngeal ring and tonsil lesions and limited diffusion in the bilateral cervical lymph nodes. **H**. Transverse T1WI with contrast showed significant enhancement of the pharyngeal ring, tonsil lesions, and bilateral cervical lymph nodes. **I**. Coronal T2WI showed enlargement of the pharyngeal ring and bilateral cervical lymph nodes

Magnetic resonance imaging (MRI) of the neck and maxillofacial region showed lesions in the nasopharynx (Fig. 1A and B C, 1D,1E, 1F), oropharynx, and tonsils (Fig. 1D, E and G H, 1I), as well as multiple enlarged lymph nodes (Fig. 1G H, 1I) in the parotid gland, retroauricular and cervical regions, necessitating differential diagnosis between lymphoma and NPC. Sagittal T1WI with contrast showed thickening with noticeable enhancement and enhancement of the nasopharynx and pharyngeal tonsils (Fig. 1A C, 1D, 1E, 1 H). Diffusion-weighted imaging showed limited diffusion in the nasopharyngeal lesion area, pharyngeal ring, tonsil lesions, and bilateral cervical lymph nodes (Fig. 1F and G). According to the MRI findings, our first impression was of a synchronous, double primary tumor, where NPC and lymphoma co-exist.

**Fig. 2:**
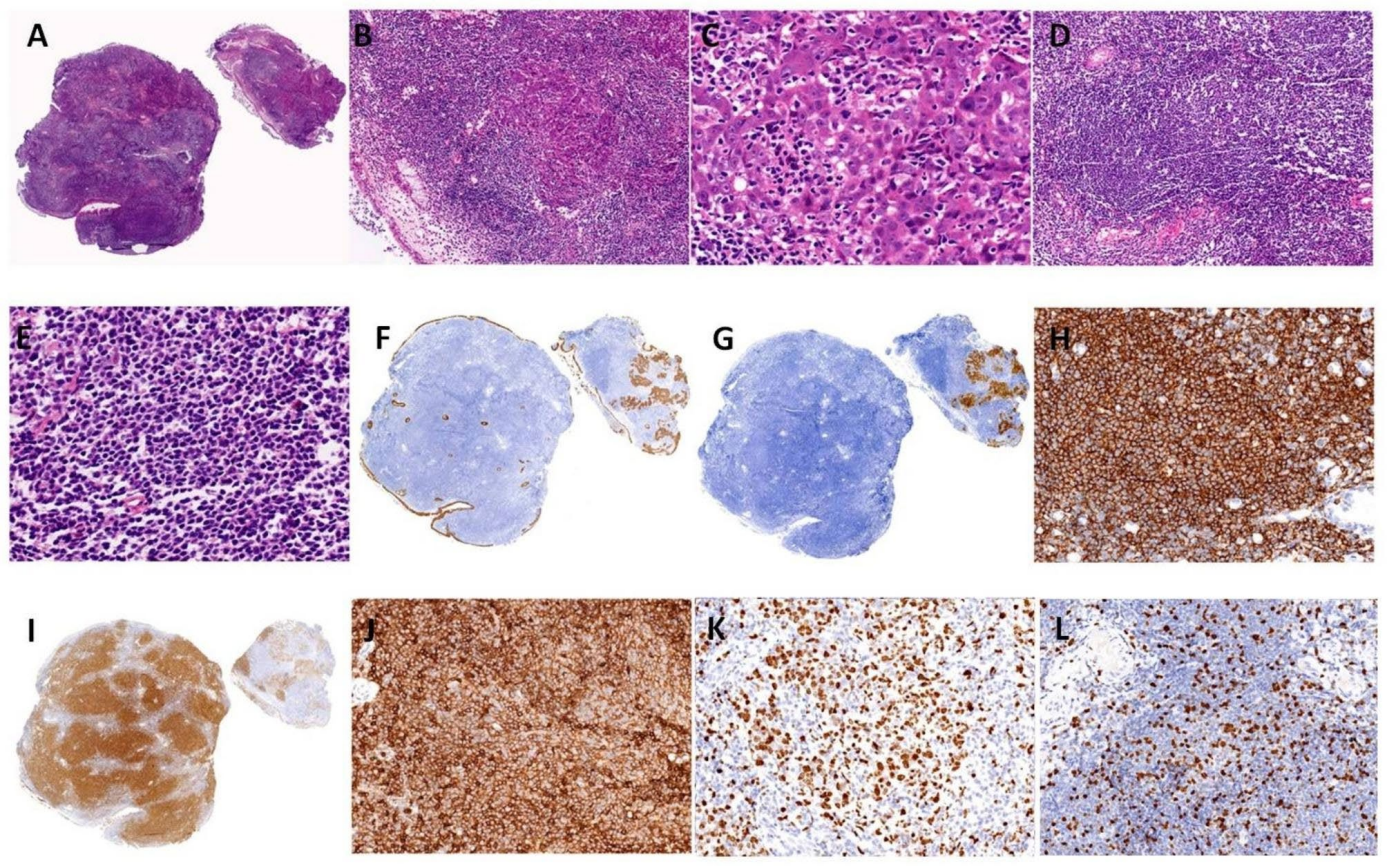
Pathological images. **A**. Two nasopharyngeal mucosal tissue specimens with abnormal structure. Left, lymphoma. Right, collision tumor of nasopharyngeal carcinoma and lymphoma. Most of the cancer was lymphoma, while a small portion was nasopharyngeal carcinoma (hematoxylin and eosin [HE], original magnification ×10). **B**. Microscopic view of the mass. Microscopic examination showed numerous atypical lymphocytes distributed in a nodular pattern, and atypical cell nests were present in focal areas of the atypical lymphoid tissue (HE, original magnification ×100). **C**. Microscopic view of the mass. Examination revealed undifferentiated non-keratinizing nasopharyngeal carcinoma of atypical cell nests (HE, original magnification ×400). **D**. Microscopic view of the mass. Examination revealed lymphoid tumor cell proliferation, suggesting mantle cell lymphoma (HE, original magnification ×100). **E**. Microscopic view of the mass. Examination revealed lymphoid tumor cell proliferation, suggesting mantle cell lymphoma, classical subtype (HE, original magnification ×400). **F**. Immunohistochemistry of nasopharyngeal carcinoma in the two small specimens of nasopharyngeal mucosal tissue was strongly positive for cytokeratin, while the atypical lymphocytes were negative (EnVision method [Dako, Glostrup, Denmark], original magnification ×10). **G**. In situ hybridization showed that the nasopharyngeal carcinoma was positive for Epstein–Barr virus (EBV)-encoded small RNAs (EBERs), while the atypical lymphocytes were negative (In situ hybridization, original magnification ×10). **H**. Immunohistochemistry of mantle cell lymphoma was strongly positive for CD20 (EnVision method, original magnification ×200). **I**. Immunohistochemistry of mantle cell lymphoma and nasopharyngeal carcinoma was positive for cyclin D1 (EnVision method, original magnification ×10). **J**. Immunohistochemistry of mantle cell lymphoma in the nasopharyngeal mucosal tissue was strongly positive for CD5 (EnVision method, original magnification ×200). **K**. Immunohistochemistry of nasopharyngeal carcinoma in the nasopharyngeal mucosal tissue was positive for Ki67 (EnVision method, original magnification ×200). **L**. Immunohistochemistry of mantle cell lymphoma in the nasopharyngeal mucosal tissue was positive for Ki67 (EnVision method, original magnification ×200)

Pathology consultation findings showed two nasopharyngeal mucosal tissue specimens measuring 0.5 × 0.4 × 0.3 cm and 0.6 × 0.6 × 0.4 cm, respectively, had an abnormal structure (Fig. 2A). Numerous atypical lymphocytes distributed in a nodular pattern (Fig. 2B). In addition to atypical lymphoid tissues, multiple focal atypical epithelial cell nests were also observed (Fig. 2B). The atypical epithelial cells contained large tumor cells with a syncytial appearance, round to oval vesicular nuclei, and large central nucleoli (Fig. 2C). The nuclei were chromatin-rich rather than vesicular, and the neoplastic cells generally had scant amphophilic or eosinophilic cytoplasm. On immunohistochemical staining, these atypical large cell nests were positive for cytokeratin (CK) (Fig. 2F), CK5/6, p63, cyclin D1 (Fig. 2I), and B-cell lymphoma 2 (Bcl-2) and negative for cluster of differentiation 3 (CD3), CD20, CD19, CD5, CD10, and Bcl-6. The MIB-1 labeling index was approximately 80% (Fig. 2K). In situ hybridization showed positivity for Epstein–Barr virus (EBV)-encoded small RNAs (EBERs) (Fig. 2G). Thus, diagnosis of the lesion was nasopharyngeal carcinoma (NPC), non-keratinizing squamous cell carcinoma, undifferentiated subtype. The morphology of the tumor with atypical lymphocytes showed monomorphic lymphoid proliferation with a vaguely nodular (Fig. 2B and D), diffuse mantle zone composed of small to medium lymphoid cells with slightly or markedly irregular nuclear contours (Fig. 2D and E). The nuclei of the atypical lymphocytes contained dispersed chromatin but inconspicuous nucleoli (Fig. 2E). In immunohistochemical staining, these atypical lymphocytes were positive for CD19, CD20 (Fig. 2H), CD5 (Fig. 2J), cyclin D1 (Fig. 2I), and Bcl-2 and negative for CD10, Bcl-6, CD3, CD30, CK (Fig. 2F), CK5/6, and p63. The MIB-1 labeling index was approximately 15% (Fig. 2L). In situ hybridization showed that the atypical lymphocytes were negative for EBERs (Fig. 2G). Fluorescence in situ hybridization of the atypical lymphocytes revealed IgH/CCND1 gene translocation. *IgH* gene rearrangement studies showed that the atypical lymphocytes were positive for the B-cell rearrangement (polymerase chain reaction + fragment analysis). Therefore, diagnosis of the lesion of atypical lymphocytes was mantle cell lymphoma (MCL), classical subtype.

**Fig. 3:**
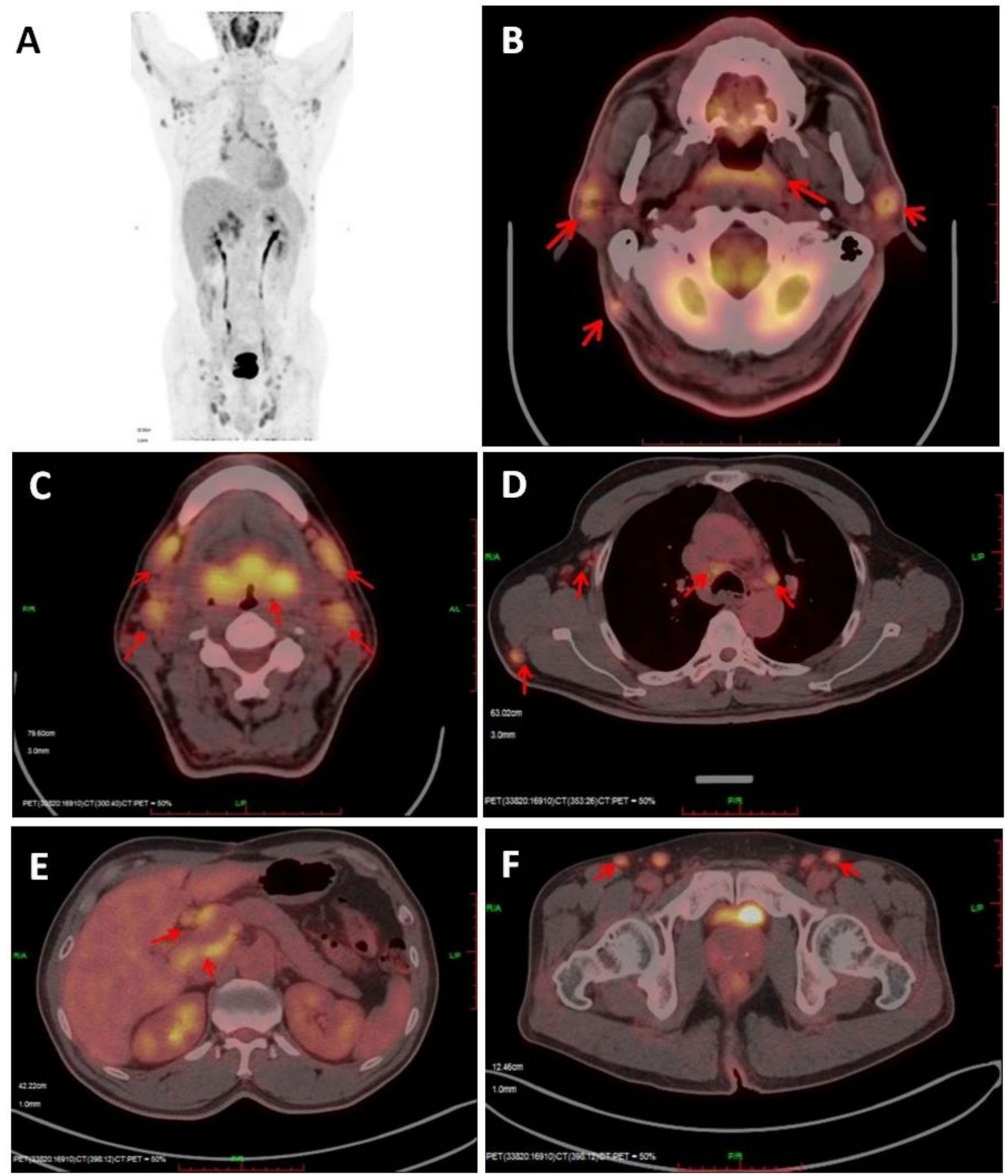
Full-body positron emission tomography computed tomography imaging. **A**. Full-body maximum intensity projection diagram. Multiple lymph nodes throughout the body, including those in the upper arm skin area, axilla, neck, mediastinum, groin and adjacent to iliac vessels, were enlarged and metabolically active. **B**. Thickening of the pharyngeal lymphoid ring and nasopharynx, enlarged neck lymph nodes, and increased glucose metabolism. **C**. Increased glucose metabolism in the pharyngeal lymphoid ring and neck lymph nodes. **D**. Active metabolism of axillary lymph nodes, mediastinal lymph nodes, and right shoulder and back lymph nodes. **E**. Active metabolism of lymph nodes in the hilar region of the liver and the peritoneal portal vena cava space. **F**. Active metabolism of the bilateral inguinal lymph nodes

Radiographs of the chest showed no evidence of metastasis in the lung. Positron emission tomography computed tomography (PET-CT) showed that the nasopharynx (Fig. 3B), oropharynx, and tonsils (Fig. 3B C) were diffusely and moderately enlarged, with multiple metabolically active lymph nodes of varying sizes throughout the body (Fig. 3A and B C, 3D, 3E, 3F). In addition, multiple small metabolically active nodules were present under the skin of the left neck, right shoulder, and left chest, as well as within the deltoid muscle of both upper limbs. Bilateral inguinal lymph nodes with active metabolism were also observed (Fig. 3F). The collision tumor of NPC and nasopharyngeal lymphoma had also been considered as a result. Multiple lymph nodes throughout the body, including five enlarged lymph nodes in the head and neck region discovered by physical examination, were infiltrated by lymphoma. The liver enlarged, but the spleen did not, and no abnormal metabolism of the liver or spleen was observed. There was no metastasis to the bone marrow, as confirmed by a bone marrow biopsy.

The combination of MRI, PET-CT, and histopathology led to diagnosing a nasopharyngeal collision tumor of NPC and primary MCL. According to the American Joint Commission on Cancer Tumor-Node-Metastasis staging system, the NPC staging is T1N0M0 [6]. The MCL was stage III according to the revised staging system for malignant lymphoma based on the Lugano classification [7, 8]. The patient’s Eastern Cooperative Oncology Group performance status (ECOG PS) was 2 [9]. The MCL International Prognostic Index (MIPI) score was calculated using the following formula: MIPI score = (0.03535 × age [years]) + 0.6978 (if ECOG PS > 1) + (1.367 × log10[LDH/ULN]) + (0.9393 × log10[WBC count]) + (0.02142 × Ki-67 [%]) [10], where ULN is the upper limit of normal. The patient’s MIPI score was 3.49, which indicated that the patient was at low risk in terms of his survival prognosis.

The patient received two courses of R-CHOP chemotherapy (rituximab, cyclophosphamide, doxorubicin, vincristine, and prednisone) followed by head and neck radiotherapy for 30 days (PGTVnx 70 Gy/30 Fr, PGTVnd-L 68 Gy/30 Fr, PGTVnd-R 68 Gy/30 Fr, PTV-1 60 Gy/30 Fr and PTV-2 54 Gy/30 Fr). After radiotherapy, the patient’s swallowing was normal, and the lesion disappeared on MRI. Follow-up physical examination, laboratory test, and imaging for over five years showed no recurrence. At the time of this writing, the patient had survived for 61 months without disease progression and was still undergoing follow-up.

## Discussion

Collision tumors of carcinoma and lymphoma often occur in the skin [4] and gastrointestinal tract [5], but they are very rare in the nasopharynx. The unusual and nonspecific presentation of collision tumors often represents a diagnostic challenge for pathologists. Collision tumors may be impossible to be accurately diagnosed on biopsy specimens if not all components of these tumors were sampled. In this case, a nasopharyngeal collision tumor of non-keratinizing squamous cell carcinoma, undifferentiated subtype, and primary MCL, classical subtype, was diagnosed. The histopathological diagnosis was precise with the help of pathological techniques, and MRI and PET-CT were used to assess the extent of the lesion. Due to the unclear mechanism of collision tumors of carcinoma-lymphoma, there is no unified guideline for their therapy. Several hypotheses have been proposed, including the mechanisms of inflammation and immunosuppression, the hypothesis of genetic factors, and the hypothesis of environmental risk factors [11]. One of the most accepted theories about the synergy between one neoplasm and another is the secondary chronic inflammatory process that solid neoplasms generate, which can trigger lymphoma. Chronic inflammatory processes such as infections (*H. Pylori*, Epstein-Barr, HIV, and hepatitis C) are known to be associated with the development of B-cell lymphoma, mainly MALT-type lymphoma. The mechanisms for this lymphoid transformation may involve mutations such as in the B cell receptor and NF-κB or the exaggerated increase of proinflammatory cytokines [12]. In this case, EBV was positive in NPC and presented a local infection in the nasopharynx, even if negative in NPL. It is speculated that the mechanism may be that in the immunosuppressive microenvironment of nasopharyngeal lymphoma, the EB virus infects the nasopharyngeal squamous epithelium, and the EB virus further integrates into the cells, initiating the mechanism of epithelial carcinogenesis. Common genetic susceptibility may also be related to collision tumors. Polymorphisms in two DNA repair genes, XRCC1 and HOGG1, have been reported to increase the risk of both lung cancer and NPC [13]. Chinese salted fish has been identified as a risk factor for NPC. This diet habit has also been suggested as a risk factor for brain cancers [14]. This case was confirmed through pathology consultation, which focused on atypical lymphocytes, and further immunohistochemistry confirmed the diagnosis. Many lymphocytes can infiltrate the stroma of malignant tumors, most of them are mixed with T and B lymphocytes. Unlike epithelial tumors, lymphocytes can exhibit various forms (varying in size and shape), from small lymphocytes to immune mother cells during reactive proliferation. Reactive proliferative cells are difficult to distinguish from lymphoma cells in morphology. Whereas the presence of lymphocytes near NPC is common, these cells are not always reactive. Therefore, the presence of dense lymphocyte infiltration should remind pathologists to carefully evaluate its morphology, immunophenotype and clonality to exclude coexisting NPLs. It is essential to closely evaluate the eventual involvement of cervical lymph nodes in the diagnosis of this type of lymphoma [15]. It is necessary to pay more attention to the atypical lymphocytes surrounding epithelial tumors and make differential diagnoses between inflammatory, reactive lymphocytes and tumor lymphoma cells, to avoid missed diagnoses and misdiagnoses.

Nasopharyngeal lymphoma (NPL) is mainly treated with chemotherapy and has a good prognosis, whereas NPC is often treated with radiotherapy as the main treatment method, supplemented by chemotherapy. NPC is the most common tumor in the nasopharynx, particularly in the southern provinces of China [16]. Therefore, NPL is prone to misdiagnosis as NPC in clinical practice. This makes the differential diagnosis of NPL and NPC is very important for guiding clinical treatment. Song et al. [17] reported that the microcirculatory blood flow and microvessel density (MVD) of NPC are more abundant than those of lymphoma. Whereas, NPL had high cellular density and few microvessels. A short time to peak (TTP) observed in NPC, but the enhancement peak (EP) and maximum contrast enhancement ratio (MCER) are higher than NPL, which was in line with the tumors’ vascularity and perfusion features on dynamic contrast-enhanced (DCE-MRI).

The key points for distinguishing lymphoma and NPC using MRI are as follows. Firstly, lymphoma exhibits diffusion limitation (diffusion-weighted imaging), while NPC does not show significant diffusion limitation. Secondly, lymphoma involves the pharyngeal ring and tonsils, while NPC does not. Thirdly, NPC is usually associated with sentinel lymph node enlargement (retropharyngeal lymph node enlargement, although retropharyngeal lymph node enlargement was not detected in this case). Fourthly, the bilateral symmetry of the lesion is a characteristic of lymphoma, while NPC can be unilateral or bilateral (mainly unilateral). Fifthly, NPC can cause nipple effusion (pharyngeal recess), but lymphoma is not always the case. NPC was more often involved in an unsymmetrical tumor with a propensity to invade widely and deeply into muscle tissue, the fat space, the neural foramen, and the skull base bone [18].

Kim et al. [19] reported that preoperative rectal MR imaging facilitated the detection of the co-existence of adenocarcinoma and lymphoma. MR imaging can be valuable in differentiating epithelial tumors from subepithelial tumors. It can be crucial for the radiologist to consider the possibility of a collision tumor and to recommend a biopsy in each cancer.

Fluorine-18 fluorodeoxyglucose (^18^ F-FDG) PET-CT also has high clinical value in the differential diagnosis of NPL and NPC [20]. Morphologically, both NPL and NPC on PET-CT images show a soft tissue thickening in the nasopharynx. NPC is usually seen as focal type, while NPL is more frequently seen as the diffuse type; additionally, NPC is more likely to involve the skull base. However, NPL is commonly associated with bilateral cervical lymph node involvement, while bilateral lymph node involvement in NPC is rare. NPL is more likely than NPC to involve the nasal cavity or paranasal sinuses and the tonsils or parotid glands. NPL occurs in the mucosal lymphoid tissue near the pharyngeal tonsils and eustachian tube tonsils in the posterior wall of the nasopharynx. It diffusely spreads along the surface of the nasopharynx wall and tends to “grow flat,” even extending to the oropharynx, nasal cavity, and sinuses; however, it generally does not invade the deep submucosal layers. Most NPLs symmetrically distribute and present as diffuse soft tissue masses, with no involvement of the bone of the skull base [21]. ^18^ F-FDG PET-CT seems helpful in staging, showing better diagnostic performance than conventional imaging and a positive impact on clinical stage, particularly for nodal lesions of MCL. ^18^ F-FDG PET-CT is also useful for evaluating the treatment response, particularly after chemotherapy and transplantation, and the metabolic response after therapy seems to have a prognostic role [22].

The treatment of collision tumors requires simultaneous treatment of the two tumor components. Nasopharyngeal carcinoma is highly sensitive to ionizing radiation. Moreover, radiotherapy is the mainstay treatment modality for nonmetastatic disease. MCL is a mature B-cell neoplasm with heterogeneous clinical behavior molecularly characterized by the constitutive overexpression of cyclin D1 and deregulation of different signaling pathways. Because the patient had a classic type of MCL, we chose chemotherapy (R-CHOP) based on the treatment of MCL combined with radiotherapy for NPC.

In a study that involved older patients with favorable early-stage Hodgkin lymphoma, two cycles of ABVD or AVD + involved-field radiotherapy were equivalent to four-course ABVD + involved-field radiotherapy in terms of efficacy and safety. In contrast, the four-course ABVD regimen showed no improvement in effectiveness but resulted in significantly increased toxicity (complete remission rates of 96%, 99%, and 88%, respectively) [23]. Our case underwent two courses of R-CHOP chemotherapy and interventional field radiotherapy, achieving complete remission for over five years. Therefore, in elderly patients with lower MIPI scores, dose-adjusted chemotherapy combined with radiotherapy may demonstrate good efficacy and safety in the treatment of NPC and MCL colliding tumors.

MCL is very sensitive to radiotherapy, even at low doses. In limited-stage MCL, radiotherapy can enable the de-escalation of systemic therapy. Radiotherapy monotherapy is a valid option for frail patients. In advanced-stage disease, radiotherapy is a very potent mode of palliation, even in heavily pretreated and chemoresistant patients. Furthermore, it can provide a respite when systemic treatment is unnecessary. Radiotherapy generally has a favorable toxicity profile, and local recurrence or distant diseases can be treated with repeated radiotherapy as needed. This effective, safe, and relatively inexpensive therapy modality has been underutilized for patients with MCL [24]. Radiotherapy provides effective and long-lasting local responses in patients with MCL and is associated with minimal toxicity. The radiation doses required for most lesions are relatively low, and responses are noticed early in the course of treatment. Radiotherapy should be considered in the early stages of recurrent, refractory, or localized MCL [25]. In the present case, its application combined with chemotherapy showed a good mid- to long-term effect in terms of disease-free progression.

## Conclusion

Correct identification and effective treatment of collision tumors are essential. We report a case involving a nasopharyngeal collision tumor of primary MCL and NPC diagnosed through pathologic consultation and immunohistochemical analyses. Clarifying the histological types by pathology and evaluating the staging and treatment plans by imaging and clinical assessments are beneficial for the managing of this rare case. Dose-adjusted chemotherapy combined with radiotherapy may show good therapeutic effects.

## Data Availability

The data supporting the results of this study can be obtained from the corresponding authors upon request.

## Abbreviations

MCL: Mantle cell lymphoma
MIPI: MCL International Prognostic Index
MRI: Magnetic resonance imaging
NPC: Nasopharyngeal carcinoma
NPL: Nasopharyngeal lymphoma
PET-CT: Positron emission tomography computed sopharyngeal carcinomatomography
R-CHOP: Rituximab, cyclophosphamide, doxorubicin, vincristine, and prednisone
